# Design and Evaluation of Voriconazole Eye Drops for the Treatment of Fungal Keratitis

**DOI:** 10.1155/2014/490595

**Published:** 2014-04-29

**Authors:** Sakshi Malhotra, Anubha Khare, Kanchan Grover, Inderbir Singh, Pravin Pawar

**Affiliations:** Chitkara College of Pharmacy, Chitkara University, Chandigarh-Patiala National Highway, Rajpura, Patiala, Punjab, 140401, India

## Abstract

Voriconazole is a novel antifungal agent with excellent broad spectrum activity commercially available for oral and intravenous administration. The purpose of this study was to prepare ophthalmic formulation of hydroxypropyl beta cyclodextrin (HP-*β*-CD) based voriconazole containing benzalkonium chloride BAK and EDTA with or without viscosity modifiers and study its permeation characteristics through freshly excised goat cornea. The results were observed that viscosity and force of bioadhesion of the voriconazole HP-*β*-CD solutions containing xanthan gum (XG) are more as compared to polyvinyl alcohol. The results revealed that voriconazole drop containing PVA provided least viscosity and higher corneal permeation of drug, while drop formulated with XG had maximum viscosity and least permeation. The HP-*β*-CD based voriconazole (1.5%) ophthalmic formulation containing xanthan gum (1.5), preserved with BAK and EDTA, could provide shelf life of 2 years. The microbiological studies showed that voriconazole ophthalmic solution containing xanthan gum shows better antifungal activity as compared to voriconazole and xanthan gum alone. Thus, it can be concluded that HP-*β*-CD based voriconazole (1.5%, pH 7.0) ophthalmic solution containing BAK and EDTA with viscosity modifier XG provided maximum precorneal residence time as compared to other viscosity modifiers and polyvinyl alcohol provided less precorneal residence time than other viscosity modifiers.

## 1. Introduction 

The inflammatory disorders of the eye parts are the manifestations of the bacterial, fungal and viral infections.* Staphylococcus aureus *(due to injury),* Pseudomonas aeruginosa *(may be due to contact lens),* Streptococcus*,* herpes simplex type I*,* varicella zoster*,* Cytomegalovirus (CMV)*,* adenovirus*,* Candida species*, and* Actinomyces israelii* are the major bacterial, viral, and fungal organisms that infect the different parts of the eyeball causing decreased vision, pain, and red eyes and may even lead to blindness [1]. Fungal keratitis is one of the major causes of ophthalmic mycosis, accounting for more than 50% of proven ophthalmic mycoses in some countries. Fungal keratitis is usually characterized by a corneal epithelial defect and inflammation of the corneal stroma. If untreated, fungal keratitis can lead to corneal scarring and vision loss. Fungal keratitis is most common in tropical regions and developing countries, where it constitutes over 50% of keratitis. The ultimate goal in the treatment of fungal keratitis is to conserve vision. This requires timely diagnosis of the infection and administration of the appropriate antifungal therapy [2]. Voriconazole is a novel second generation triazole derivative of fluconazole with excellent broad spectrum activity commercially available for oral and intravenous administration. Systemic administration of voriconazole is associated with adverse effects including cardiac arrhythmias, visual disturbances, acute renal impairment, and hepatic abnormalities. Voriconazole has broad* in vitro* antifungal activity against yeasts and molds, including a wide range of less common pathogens. Voriconazole possesses fungicidal* in vitro* activity against all* Aspergillus* species, molds such as* Scedosporium* species, and* Fusarium* species and is highly potent against fluconazole-resistant* Candida* species including* Candida krusei*,* Candida glabrata*, and* Candida albicans*. The 90% minimum inhibitory concentrations (MIC90) of voriconazole are considerably less than that of fluconazole. Voriconazole inhibits cytochrome P450-dependent 14*α* sterol demethylase enzyme which is responsible for inhibiting and disrupting fungal cell membrane synthesis, resulting in depletion of ergosterol [3].

Voriconazole ophthalmic drops have not yet been marketed but promising results obtained with the use of voriconazole drops prepared by reconstitution of Vfend powder in several clinical studies have demonstrated the need for topical formulations [4, 5].

Commonly, all the ophthalmic formulations have been administered to the eye as aqueous solutions. About 90% of the dose applied topically from such solutions is lost due to precorneal losses (nosolacrimal drainage) results poor availability [6]. Voriconazole is a lipophilic drug with a low pH dependent aqueous solubility (maximum 2.7 mg/mL at pH 1.2). The hydrophilic character of stroma in cornea restricts the permeation of lipophilic drug molecules through cornea. Commercially available Vfend (powder for reconstitution) for intravenous administration uses sulfobutyl ether-*β*-cyclodextrin (SBE-*β*-CD) to enhance the solubility of voriconazole [7].

Hydroxypropyl-*β*-cyclodextrin (HP-*β*-CD), a cyclic oligosaccharide with outer hydrophilic surface and a lipophilic cavity, is capable of forming inclusion complexes with many lipophilic drugs. Solubility enhancement studies of indomethacin conducted using SBE-*β*-CD and HP-*β*-CD have revealed the better potential of HP-*β*-CD as a solubility enhancing agent [8]. Aqueous ophthalmic solutions of poorly soluble drugs methazolamide and disulfiram with HP-*β*-CD and HPMC have been formulated for the topical treatment of glaucoma and cataract [9]. Ciprofloxacin ophthalmic formulations prepared using HP-*β*-CD demonstrated better stability and biological activity than the ophthalmic solution without HP-*β*-CD [10].

The purpose of this study was to formulate voriconazole aqueous drops using 2-hydroxypropyl-*β*-cyclodextrin (HP-*β*-CD) and to determine the effect of preservative and viscosity modifiers such as sodium alginate, poly vinyl alcohol, xanthan gum, guar gum, sodium carboxymethyl cellulose, gelrite, an anionic polymers, and chitosan, a cationic polymer on the transcorneal permeation of voriconazole through freshly excised goat corneas. The release of voriconazole from voriconazole-HP-*β*-CD based ophthalmic formulation was studied by conducting microbiological assay studies and stability studies of all ophthalmic formulations containing viscosity modifiers at both room and accelerated storage conditions were performed.

## 2. Materials and Methods

### 2.1. Material

Voriconazole and hydroxypropyl-*β*-cyclodextrin were obtained as gift samples from Matrix Laboratories, Hyderabad (India), mucin from porcine stomach type II, sodium alginate, chitosan, sodium carboxymethyl cellulose, poly vinyl alcohol, xanthan gum, guar gum, and gelrite were purchased from Sigma Aldrich. Hi media Sabouraud Dextrose Agar was obtained from Deep Scientific laboratories, Chandigarh (India). Freeze dried strains* Candida albicans* (MTCC 227) and* Aspergillus fumigatus* (MTCC 2544) were obtained from MTCC, IMTECH Chandigarh (India). Fresh whole eyeballs of goat were obtained from local butcher's shop (Zirakpur, Punjab, India) within one hour of slaughtering of animal. All other chemicals used were of analytical reagent grade.

### 2.2. Methods

#### 2.2.1. Preparation of Test Solutions

#### 2.2.2. Voriconazole Ophthalmic Solutions (1.5% w/v, pH 7.0) Containing Different Preservatives

Voriconazole (150 g) and HP-*β*-CD (2.5 g) were dissolved in sufficient distilled water followed by the addition of sodium chloride (0.687 g) with constant stirring by using magnetic stirrer. To this solution benzalkonium chloride (BAK, 0.01% w/v) or benzyl alcohol (BA, 0.05% w/v) or thiomersal (THM, 0.005% w/v) or phenyl mercuric acetate (PMA, 0.002% w/v) or phenyl mercuric nitrate (PMN, 0.002% w/v) or disodium edetate (EDTA, 0.01% w/v) or a combination of BAK (0.01% w/v) and EDTA (0.01% w/v) was added and pH of the solutions were adjusted to 7.0 using 0.1 N HCl or 0.1 N NaOH. The final volume of each solution was made up to 10 mL with distilled water.

#### 2.2.3. Voriconazole Ophthalmic Solutions (1.5% w/v, pH 7.0, BAK (0.01%) and EDTA (0.01%)) Containing Different Viscosity Enhancing Agents

Voriconazole (150 mg) and HP-*β*-CD (2.5 g) were dissolved in sufficient distilled water followed by the addition of sodium chloride and BAK (0.01% w/v) and EDTA (0.01% w/v). To this solution, different quantities of viscosity modifiers or enhancing agents were added, that is, sodium alginate (2.0% w/v) or chitosan (0.2% w/v) or poly vinyl alcohol (1.4%) or sodium carboxymethyl cellulose (2.5%) or xanthan gum (1.5%) or guar gum (1.5%) or gelrite (0.5%) was added and pH of the solutions were adjusted to 7.0 using 0.1 N HCl or 0.1 N NaOH. The final volume of each solution was made up to 10 mL with distilled water.

### 2.3. Evaluation

#### 2.3.1. Measurement of Viscosity

The effect of viscosity modifiers in the voriconazole ophthalmic solutions containing different viscosity modifiers was determined by Brookfield viscometer (Brookfield DV + Pro, Brookfield Engineering Laboratories, Middleboro, MA, USA). Viscosity modifiers can affect the permeation by modifying the corneal contact time.

#### 2.3.2. *In Vitro* Transcorneal Permeability

Drug permeation studies were carried out by using freshly excised goat cornea. Goat whole eyeballs were transported from the local butcher shop to the laboratory in cold (4°C) normal saline within 1 hour of slaughtering of the animal. The cornea was carefully excised along with 2 to 4 mm of surrounding sclera tissue and was washed with cold normal saline till the washing was free from proteins. The receptor compartment of an all-glass modified Franz diffusion cell was filled with 10 mL freshly prepared normal saline solution (pH 7.0), and all air bubbles were expelled from the compartment. Freshly excised cornea was fixed between clamped donor and receptor compartments in such a way that its epithelial surface faced the donor compartment. The corneal area available for diffusion was 0.75 cm^2^. An aliquot (1 mL) of test solution was placed on the cornea and the opening of the donor cell was sealed with a glass cover slip. The receptor fluid was kept at 37°C with constant stirring using a Teflon-coated magnetic stir bead. Permeation study was continued for 120 minutes, and samples were withdrawn from receptor and analyzed for voriconazole content by measuring absorbance at 257 nm in a spectrophotometer (UV-Vis spectrophotometer 2701 A Systronics, Mumbai, India). Results were expressed as amount permeated and percentage permeation or* in vitro* ocular availability. The apparent corneal permeability coefficient and percentage permeation (or* in vitro* ocular availability) was calculated as follows:

(1) Papp(Apparent  corneal  permeability)  =(Qt)×1A·Co·60,

where *Q*/*t* (*μ*g/min) is the flux across the corneal tissue, *A* is the area of diffusion (cm^2^), *C*_*o*_ (*μ*g/cm^3^) is the initial concentration of drug in donor compartment, and 60 is taken as the factor to convert minute to second:

(2) %  In  vitro  permeation=Amount  of  drug  permeated  in  receptorInitial  amount  of  drug  in  donor×100.

#### 2.3.3. Corneal Hydration (%)

At the end of each permeation study, the cornea (freed from adhering sclera) was weighed and soaked in 1 mL methanol and dried overnight at 90°C and reweighed.

(3) Corneal  hydration  =(Initial  weight−Final  weight)Initial  weight  ×100.

#### 2.3.4. Bioadhesive Strength

Mucin-polymer bioadhesive strength was determined by a simple viscometric method described by Hassan and Gallo [11]. Viscosities of 15% (w/v) porcine gastric mucin dispersions in normal saline were determined by a Brookfield viscometer in the absence (*η*_*m*_) or presence (*η*_*t*_) of different formulations at a shear rate of 100 rpm at 37°C. Viscometric measurements were performed after exactly 3 min of applying the shear force to be homogeneously distributed throughout the sample. Viscosity component of bioadhesion (*η*_*b*_) was calculated from the equation, *η*_*t*_ = *η*_*m*_ + *η*_*p*_ + *η*_*b*_, where *η*_*p*_ is the viscosity of corresponding pure polymer solution. The force of bioadhesion (*F*) was calculated from the equation, *F* = *η*_*b*_ · *σ*, where *σ* is the rate of shear/sec.

### 2.4. Stability Studies

Stability studies were performed on voriconazole ophthalmic solutions according to ICH guidelines. All ophthalmic formulations were stored in closed amber glass bottles and kept in humidity chamber at accelerated (40 ± 2°C, 75 ± 5% RH) and room temperature conditions (30°C ± 2°C, 65% RH ± 5% RH).

Samples were withdrawn at time interval 0 days, 3 weeks, 6 weeks, 3 months, and 6 months. The samples were evaluated for their drug content and pH. The degradation rate constant (*K*_calc_), shelf life (*t*_90_), and initial concentrations providing two years shelf life (Int_calc_) were determined [12].

### 2.5. Antifungal Studies

Sabouraud dextrose agar (mycological peptone 10 mg, dextrose 10 g) was used to prepare the medium. Sabouraud dextrose agar was used in quantity of 65 g and dissolved by heating in one liter of distilled water with frequent agitation and boiled for 1 min to completely dissolve the powder. Autoclave the media at 121°C for 15 min. The media were poured into 200 mm diameter plastic plates and left to solidify for 15 min. Wells of 10 mm diameter were punched out using a steel borer. The antifungal activity of voriconazole containing different viscosity modifier was measured by plate microbioassay (agar cup diffusion) method.* Candida albicans *and* Aspergillus fumigatus* were inoculated onto the agar surface by streaking. An aliquot of 50 *μ*L of test samples was placed in separate 10 mm wells in triplicate after appropriate dilution with distilled water. The plates were left for 30 min and then incubated at 25°C for 24 hours. The diameters of zone of inhibition for* Candida albicans* and* Aspergillus fumigatus* were measured after 24 hours and 120 hours, respectively.

### 2.6. Statistical Analysis

All values presented in this study are average of triplicate experiments for the same time points. Differences in the* in vitro* permeability profile of vorioconazole under different conditions were tested statistically using one-way analysis of variance (ANOVA) followed by Dunnett's test at different level of significance (see Tables 1, 2, and 3).

## 3. Results and Discussion

### 3.1. Viscosity Modifiers

**Figure 1:**
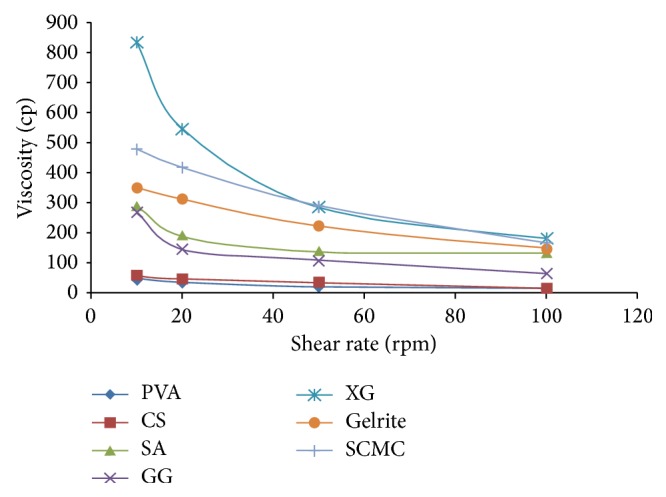
Comparative rheological studies of voriconazole ophthalmic formulations containing different viscosity modifiers. Values are mean ± SE of 3 in each group. PVA refers to polyvinyl alcohol, CS refers to chitosan, GG refers to guar gum, SA refers to sodium alginate, SCMC refers to sodium carboxymethyl cellulose, and XG refers to xanthan gum and gelrite.

All the ophthalmic formulations containing different viscosity modifiers show pseudo plastic behavior, that is, high viscosity at low shear rate and low viscosity at high shear rate. The optimized formulation, that is, xanthan gum, shows high viscosity than other viscosity modifiers. Hence, precorneal residence time of ophthalmic formulation containing xanthan gum is more. This occurs due to hydrogen bonding and polymer entanglement. Highly ordered network of entangled, stiff molecules results in high viscosity at low shear rate [13]. At low shear rates, solutions of xanthan gum have more viscosity as compared to guar gum, sodium alginate, sodium carboxymethyl cellulose, polyvinyl alcohol, gelrite, and chitosan as shown in Figure 1 [14].

### 3.2. Bioadhesive Strength

The force of bioadhesion between the voriconazole solutions containing sodium alginate, chitosan, polyvinyl alcohol, gelrite, xanthan gum, guar gum, and sodium carboxymethyl cellulose at different concentrations with mucin is shown in Table 1. Voriconazole ophthalmic solutions containing chitosan (0.2% w/v), sodium alginate (0.4% w/v), polyvinyl alcohol (1.4%), xanthan gum (1.5%), sodium carboxymethyl cellulose (2.5%), guar gum (1.5%), and gelrite (0.5%) exhibited bioadhesive strength of 52.61 ± 0.63 dyne/cm^2^, 64.38 ± 0.25 dyne/cm^2^, 47.88 ± 0.25 dyne/cm^2^, 84.33 ± 0.16 dyne/cm^2^, 78.33 ± 0.25 dyne/cm^2^, 57.90 ± 0.44 dyne/cm^2^, and 72.16 ± 0.33 dyne/cm^2^. Therefore, xanthan gum (1.5% w/v) produced a higher force of bioadhesion as compared to other viscosity modifiers as shown in Table 1. The study demonstrated the bioadhesive strength of various polymers on corneal surface. The polymers hydroxypropylmethylcellulose, carboxymethylcellulose sodium, Eudragit type E/RL/RS, Carbopol ETD 2020, and Carbopol 934 were formulated with drug, ketorolac tromethamine. This results in good bioadhesion with improved corneal penetration [15].

### 3.3. *In Vitro* Transcorneal Permeation Studies

Preservatives have been acting as absorption promoters for different drugs when they act on corneal membrane. It helps in preserving the sterility of the eye drops and stability of drug dispensed in multiusage containers. The effect of addition of different preservatives in voriconazole (1.5% w/v, pH 7.0) HP-*β*-CD based aqueous ophthalmic solution through freshly excised goat cornea is depicted in Table 2. The addition of BAK (0.01% w/v) and EDTA (0.01%) to voriconazole (1.5% w/v, pH 7.0) drops increased the apparent corneal permeability coefficient (*P*_app_) by about 1.2 and 1.3 times from 0.58 (×10^−6^) ± 0.006 to 2.06 (×10^−6^) ± 0.067 cm/s (*P* < 0.001) and 2.21 (×10^−6^) ± 0.025 cm/s (*P* < 0.0001), respectively. A combination of BAK (0.01% w/v) and EDTA (0.01% w/v) in voriconazole ophthalmic solutions provide higher apparent permeability coefficient, that is, 30.72 (×10^−6^) ± 0.055 cm/s, which is statistically significant (*P* < 0.001). EDTA is used as a buffering agent and to preserve the stability of eye drops due to its ability to chelate heavy metals. It has been found that calcium removal from endothelial and epithelial layers of cornea enhances the permeability of the layers. EDTA can form chelates with calcium ions present in the epithelial and endothelial corneal layers and induce ultrastructural changes in the corneal epithelium, resulting in increased water influx and decreased overall lipophilic characteristics [16, 17].

BAK, a cationic surfactant, is used to increase corneal permeability even at very low concentrations by reducing surface tension at the corneal interface and also by membrane disruption. The BAK molecules intercalate in the apical cell layer and form channels for increased drug transport for longer time period [18]. Therefore, it has the potential to significantly enhance the penetration of drugs. Organomercurials (thiomersal, phenyl mercuric nitrate, and phenyl mercuric acetate) react with sulfhydryl groups in cornea, leading to an increase in membrane permeability and alterations of membrane transport systems. The study has shown similar results with the addition of thiomersal, PMN, and PMA. Thiomersal is reported to cause structural and functional changes in endothelium on prolonged exposure [19].

**Figure 2:**
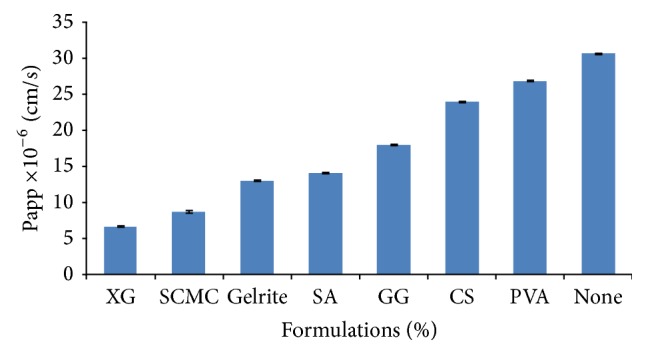
Comparative transcorneal permeability profile of different voriconazole ophthalmic solutions (1.5%, 7.0), that is, none (without viscosity modifiers) and voriconazole ophthalmic solutions containing viscosity modifiers through freshly excised goat cornea. Values are mean ± SE of 3 corneas in each group.

The effect of highest concentration of all viscosity modifiers on the* in vitro *permeation and apparent corneal permeability through freshly excised goat corneas is shown in Table 3 and Figure 2. Results indicate that the permeation of voriconazole (0.5% w/v, pH 7.0) from drops containing either XG, SCMC, gelrite, SA, GG, CS, or PVA was significantly (*P* < 0.01) less than that observed with formulation containing no viscosity agent (none). As the viscosity of drop increased, the permeation of voriconazole decreased. Voriconazole drop containing PVA provided least viscosity and higher corneal permeation of drug, while drop formulated with XG had maximum viscosity and least permeation. Among all the formulations, the formulation containing xanthan gum produced maximum viscosity, that is, 180.28 (cp), at 100 rpm as compared to other formulations. The study demonstrates that the increase in viscosity of eye drops would decrease diffusion coefficient of the drug and same could result in reduced permeation [20]. Ophthalmic formulations containing xanthan gum (1.5%) produced significantly (*P* < 0.01) lesser apparent corneal permeability and permeation, as compared with other formulations. The voriconazole formulations containing xanthan gum showed lesser as compared to other formulations.

### 3.4. Corneal Hydration (%)

The corneal hydration level of normal mammalian cornea is between 75% and 80% [21]. The increase in drug concentration, addition of preservatives, and/or viscosity modifiers in ophthalmic solutions did not show any corneal damage as the corneal hydration value for all corneas remained in the normal range of 75% to 80%.

### 3.5. Stability Studies

The results of accelerated and long term stability studies conducted on voriconazole ophthalmic solution containing viscosity modifiers are shown in Tables 4 and 5, respectively, and were examined for their pH and drug content. The results indicated that there is no change in pH. All the formulations showed more than 90% of drug content during both accelerated and room temperature storage conditions. The degradation of voriconazole ophthalmic solution followed first-order kinetics. The *K*_calc_ and *t*_90_ values of all the formulations at room temperature are shown in Table 5. The degradation rate constants (*K*_calc_) and shelf life (*t*_90_) at room temperature for all ophthalmic formulations range between 1.10 to 2.60 day^−1^ and 404 to 929 days, respectively, as shown in Table 4. The *K*_calc_ and *t*_90_ values suggest that most of the formulations will not provide 2 years of shelf life (*t*_90_) of the product and might need some overages resulting in higher initial drug concentration, and the same has been shown in last column of Table 5. Among all the formulations except formulation containing xanthan gum shows better stability. Among all the formulations except voriconazole ophthalmic solution containing xanthan gum would require more overages to ensure a shelf life of 2 years at room temperature.

### 3.6. Antifungal Studies

**Figure 3:**
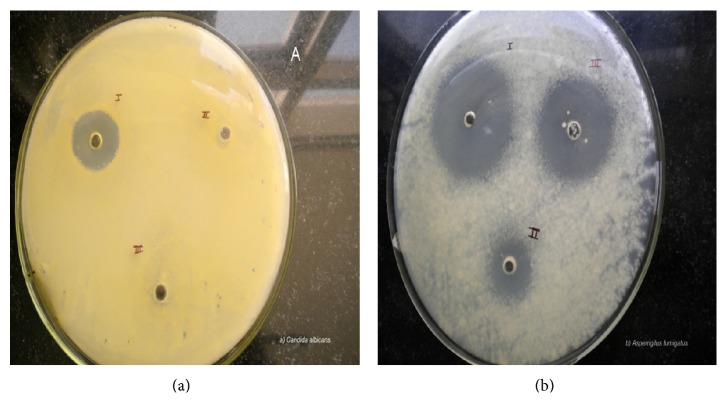
Microbiological studies using I. Voriconazole with HP-*β*-CD ophthalmic solution containing viscosity modifier II. Solution containing voriconazole with HP-*β*-CD III. Solution containing only viscosity modifier against (a)* Candida albicans* and (b)* Aspergillus fumigatus* by agar diffusion method after 24 and 120 hours, respectively.

The microbiological assay studies were conducted using agar diffusion method. The results indicate that the voriconazole formulation with or without viscosity modifier significantly inhibit the growth of* Candida albicans* and* Aspergillus fumigates *as compared than the formulation containing only viscosity modifier. The mean diameter of zone of inhibition with* Candida albicans* and* Aspergillus fumigatus *are depicted in Tables 6 and 7 and Figure 3. The lack of growth inhibition in the presence of viscosity modifier alone revealed that the fungal growth inhibition can be attributed to the drug. Voriconazole exhibited superior antifungal activity against* Aspergillus fumigatus* than* Candida albicans*. Therefore, HP-*β*-CD promotes the release of drug through cornea as evident from the microbiological assay. HP-*β*-CD based voriconazole ophthalmic solution containing xanthan gum as viscosity modifier inhibited the fungal growth. It can be hypothesized that xanthan gum deposits over the precorneal surface and creates a concentration gradient for the drug molecules to permeate through cornea and provide more precorneal residence time. The prolonged residence time of voriconazole ophthalmic solution containing xanthan gum occurs due to the increased viscosity of xanthan gum (1.5% w/v).

## 4. Conclusions

On the basis of results available, it can be concluded that HP-*β*-CD (2.5 gm) based voriconazole (1.5% w/v) ophthalmic formulation containing BAK (0.01% w/v) and EDTA (0.01% w/v) provides maximum* in vitro* transcorneal permeate of voriconazole through goat cornea. The transcorneal permeation of voriconazole formulation containing viscosity modifier produce less permeation of drugs as compared to formulation without viscosity modifier. Increasing the viscosity of drops by addition of viscosity modifier however reduces the permeation of voriconazole. The HP-*β*-CD based voriconazole (1.5%) ophthalmic formulation containing xanthan gum (1.5), preserved with BAK and EDTA, could provide shelf life of 2 years. The microbiological studies showed that voriconazole ophthalmic solution containing xanthan gum shows better antifungal activity as compared to voriconazole and xanthan gum alone. Thus, it can be concluded that HP-*β*-CD based voriconazole (1.5%, pH 7.0) ophthalmic solution containing BAK (0.01% w/v) and EDTA (0.01% w/v) with viscosity modifier xanthan gum (1.5% w/v) provided maximum precorneal residence time as compared to other viscosity modifiers and polyvinyl alcohol (1.4% w/v) provided less precorneal residence time than other viscosity modifiers.

## Figures and Tables

**Table 1:** Effect of the addition of different polymers to voriconazole ophthalmic solutions at different concentrations on viscosity of solutions, bioadhesion component, and force of bioadhesion.

Voriconazole ophthalmic solutions (1.5% w/v, pH 7.0)	Viscosity of solutions at 100 rpm (cP)	Viscosity of bioadhesive component at 100 rpm (cP)	Force of bioadhesion (dyne/cm^2^)
Control	3.01 ± 0.02	165.20 ± 0.200	19.81 ± 0.333
PVA 1.4%	13.10 ± 0.100^††^	166.63 ± 0.378^††^	47.88 ± 0.254^†††^
CS 0.20%	15.23 ± 0.246^††^	192.33 ± 0.152^††^	52.61 ± 0.631^†††^
GG 1.5%	62.66 ± 0.321^††^	247.70 ± 0.264^††^	57.90 ± 0.441^†††^
SA 2.00%	132.50 ± 0.500^††^	321.43 ± 0.152^††^	64.38 ± 0.254^†††^
Gelrite 0.5%	147.10 ± 0.100^††^	340.70 ± 0.200^††^	72.16 ± 0.333^†††^
SCMC 2.5%	167.80 ± 0.100^††^	365.13 ± 0.152^††^	78.33 ± 0.254^†††^
XG 1.5%	180.20 ± 0.264^††^	381.10 ± 0.100^††^	84.33 ± 0.166^†††^

^*^PVA refers to polyvinyl alcohol, CS refers to chitosan, GG refers to guar gum, SA refers to sodium alginate, SCMC refers to sodium carboxymethyl cellulose, and XG refers to xanthan gum.

Values are mean ± SE of 3 solutions viscosity in each group.

^†^Statistically significant difference at *P* < 0.05; ^††^statistically significant difference at *P* < 0.01; ^†††^statistically significant difference at *P* < 0.001 from control (voriconazole-HP-*β*-CD based solutions 1.5%, pH 7.0) as determined by one-way ANOVA followed by Dunnett's test.

**Table 2:** Effect of preservatives on the transcorneal permeation of voriconazole from ophthalmic solutions through freshly excised goat cornea.

Preservatives	Amount of drug permeated in 120 min (mg)	% *in vitro* permeation	*P*_app_ × 10^−6^ (cm/s)	Corneal hydration (%)
Control	0.047 ± 0.001	0.314 ± 0.003	1.73 ± 0.017	79.23 ± 0.30
Benzyl alcohol	0.046 ± 0.001	0.308 ± 0.002	1.70 ± 0.013	79.35 ± 0.24
PMA	0.052 ± 0.001^†^	0.349 ± 0.003^†^	1.93 ± 0.071^†^	78.33 ± 0.61
PMN	0.054 ± 0.001^††^	0.358 ± 0.003^††^	1.98 ± 0.071^††^	79.07 ± 1.76
Thiomersal	0.058 ± 0.001^†††^	0.384 ± 0.007^†††^	2.12 ± 0.021^†††^	79.96 ± 0.57
BAK	0.056 ± 0.002^††^	0.373 ± 0.012^†††^	2.06 ± 0.067^†††^	79.53 ± 0.03
EDTA	0.060 ± 0.001^†††^	0.400 ± 0.005^†††^	2.21 ± 0.025^†††^	79.37 ± 0.26
BAK + EDTA	0.317 ± 0.001^††^	2.120 ± 0.003^††^	30.72 ± 0.055^†††^	77.88 ± 0.15

Values are mean ± SE of 3 corneas in each group.

^†^Statistically significant difference at *P* < 0.05; ^††^statistically significant difference at *P* < 0.01; ^†††^statistically significant difference at *P* < 0.001 from control (voriconazole 1.5%, pH 7.0 HP-*β*-CD based solution) as determined by one-way ANOVA followed by Dunnett's test.

**Table 3:** Effect of different viscosity modifiers on the *in vitro* transcorneal permeation of voriconazole from ophthalmic solution through freshly excised goat cornea.

Formulations	Viscosity (cp) at 100 rpm	Amount of drug permeated in 120 mins	% *in vitro* permeation	*P*_app_ × 10^−6^ (cm/s)	Corneal hydration
None	0.98	0.317 ± 0.001^††^	2.12 ± 0.003^††^	30.72 ± 0.055^††^	77.88 ± 0.15
PVA 1.4%	05.43	0.287 ± 0.001^††^	1.918 ± 0.003^††^	26.91 ± 0.051^††^	77.63 ± 1.40
CS 2%	15.23	0.256 ± 0.001^††^	1.71 ± 0.003^††^	23.99 ± 0.050^††^	77.71 ± 1.48
GG 1.5%	62.66	0.192 ± 0.001^††^	1.281 ± 0.003^††^	17.97 ± 0.050^††^	79.36 ± 0.35
SA 2%	132.55	0.151 ± 0.001^††^	1.01 ± 0.001^††^	14.14 ± 0.039^††^	79.93 ± 0.38
Gelrite 0.5%	147.10	0.139 ± 0.001^††^	0.92 ± 0.003^††^	13.03 ± 0.055^††^	78.92 ± 0.47
SCMC 2.5%	167.80	0.092 ± 0.001^††^	0.61 ± 0.003^††^	8.75 ± 0.195^††^	76.68 ± 1.82
XG 1.5%	180.28	0.071 ± 0.001	0.48 ± 0.002	6.79 ± 0.050	77.53 ± 0.47

Values are mean ± SE of 3 solutions viscosity in each group.

^†^Statistically significant difference at *P* < 0.05; ^††^statistically significant difference at *P* < 0.01; ^†††^statistically significant difference at *P* < 0.001 from control (voriconazole-HP-*β*-CD based solutions 1.5%, pH 7.0) as determined by one-way ANOVA followed by Dunnett's test.

**Table 4:** Stability profile of voriconazole ophthalmic solutions containing different viscosity modifiers under accelerated conditions (40°C ± 2°C, 75% RH ± 5%).

Formulations	Drug content (%)					pH				
	0 D	3 W	6 W	3 M	6 M	0 D	3 W	6 W	3 M	6 M
Control	100 ± 0.01	97.01 ± 0.04	97.12 ± 0.02	97.00 ± 0.02	94.23 ± 0.09	7.0	6.8	6.8	6.8	6.8
XG	100 ± 0.00	98.68 ± 0.01	98.02 ± 0.01	97.22 ± 0.00	97.10 ± 0.00	7.0	7.0	6.9	6.9	6.9
SCMC	100 ± 0.00	98.58 ± 0.01	97.89 ± 0.01	97.13 ± 0.00	96.78 ± 0.01	7.0	7.0	6.9	6.9	6.8
Gelrite	100 ± 0.00	98.52 ± 0.01	97.69 ± 0.00	97.10 ± 0.00	96.60 ± 0.01	7.0	6.9	6.9	6.8	6.8
NA	100 ± 0.00	98.45 ± 0.00	97.87 ± 0.01	97.24 ± 0.00	96.48 ± 0.00	7.0	6.9	6.8	6.8	6.8
GG	100 ± 0.01	98.28 ± 0.00	97.92 ± 0.01	97.56 ± 0.00	96.36 ± 0.01	7.0	6.9	6.8	6.8	6.8
CS	100 ± 0.01	97.96 ± 0.00	97.44 ± 0.00	96.88 ± 0.01	95.97 ± 0.00	7.0	6.8	6.8	6.8	6.7
PVA	100 ± 0.01	97.44 ± 0.00	96.97 ± 0.00	96.47 ± 0.01	95.69 ± 0.01	7.0	6.8	6.7	6.7	6.7

Values are mean of ± SD (*n* = 3). D: days; W: weeks; M: months.

**Table 5:** Stability profile of voriconazole ophthalmic solutions containing different viscosity modifiers under room conditions (30°C ± 2°C, 65% RH ± 5% RH).

Formulations	Drug content (%)					pH					*K*_calc_ (days^−1^ × 10^4^)	*t*_90_ days	Int_calc_ for 2 years
	0 D	3 W	6 W	3 M	6 M	0 D	3 W	6 W	3 M	6 M			
Control	100 ± 0.01	98.00 ± 0.01	97.70 ± 0.02	97.11 ± 0.03	95.12 ± 0.01	7.0	6.9	6.7	6.6	6.3	2.59	404	109.7
XG	100 ± 0.01	99.74 ± 0.01	99.21 ± 0.00	98.90 ± 0.00	97.98 ± 0.00	7.0	7.0	7.0	6.9	6.9	1.13	929	104.2
SCMC	100 ± 0.00	99.73 ± 0.00	99.11 ± 0.00	98.77 ± 0.00	97.31 ± 0.01	7.0	6.9	6.9	6.9	6.9	1.51	694	105.5
Gelrite	100 ± 0.00	99.40 ± 0.00	98.83 ± 0.00	98.58 ± 0.01	97.18 ± 0.01	7.0	6.9	6.9	6.8	6.8	1.58	660	105.8
SA	100 ± 0.00	99.23 ± 0.01	98.74 ± 0.01	98.27 ± 0.01	97.11 ± 0.01	7.0	6.9	6.9	6.8	6.8	1.62	645	106.0
GG	100 ± 0.00	99.10 ± 0.00	98.66 ± 0.01	98.11 ± 0.01	96.97 ± 0.00	7.0	6.9	6.8	6.8	6.8	1.71	616	106.3
CS	100 ± 0.00	99.00 ± 0.00	98.43 ± 0.01	98.10 ± 0.01	96.24 ± 0.00	7.0	6.8	6.8	6.8	6.8	2.12	493	107.8
PVA	100 ± 0.00	98.05 ± 0.01	97.76 ± 0.01	97.48 ± 0.01	95.83 ± 0.00	7.0	6.7	6.7	6.7	6.7	2.36	444	108.7

^*^Values are mean of ± SD (*n* = 3); D: days; W: weeks; M: months; *K*_calc_: calculated first-order degradation rate constant; *t*_90_: time to reach 90% of initial drug concentration; Int_calc_: calculated initial drug concentration for shelf life (*t*_90_) of 2 years.

**Table 6:** A comparative study of antifungal activity of voriconazole solutions with viscosity modifier (xanthan gum), xanthan gum alone, and HP-*β*-CD based voriconazole ophthalmic solution against *Candida  albicans*.

S. number	Solution	Mean of diameter of zone of inhibition (mm) ± SE	Range of zone size (mm)	Coefficient of variance (%)
I	Voriconazole + cyclodextrin complex (HP-*β*-CD)	12.05 ± 0.04	12.01–12.12	0.33
III	Viscosity modifier (xanthan gum 1.5%)	10.04 ± 0.03	10.01–10.14	0.30
IV	Voriconazole + HP-*β*-CD + xanthan gum (1.5% w/v)	32.23 ± 0.15	32.20–32.30	0.48

**Table 7:** A comparative study of antifungal activity of voriconazole solutions with viscosity modifier (xanthan gum), xanthan gum alone, and HP-*β*-CD based voriconazole ophthalmic solution against *Aspergillus  fumigatus*.

S. number	Solution	Mean of diameter of zone of inhibition (mm) ± SE	Range of zone size (mm)	Coefficient of variance (%)
I	Voriconazole + cyclodextrin complex (HP-*β*-CD)	12.07 ± 0.02	12.01–12.12	0.17
III	Viscosity modifier (xanthan gum 1.5%)	10.04 ± 0.02	10.01–10.14	0.19
IV	Voriconazole + HP-*β*-CD + xanthan gum (1.5% w/v)	68.37 ± 0.55	68.31–68.45	0.81

## References

[1] Henderer J. D., Raprano C. J., Brunton L. L., Lazo J. S., Parker K. L. (2005). Ocular pharmacology. *Goodman & Gilman’s the Pharmacological Basis of Therapeutics*.

[2] Manzouri B., Wyse R. K. H., Vafidis G. C. (2001). Pharmacotherapy of fungal eye infections. *Expert Opinion on Pharmacotherapy*.

[3] Johnson L. B., Kauffman C. A. (2003). Voriconazole: a new triazole antifungal agent. *Clinical Infectious Diseases*.

[4] Lau D., Fedinands M., Leung L. (2008). Penetration of voriconazole, 1%, eyedrops into human aqueous humor: a prospective open-label study. *Archives of Ophthalmology*.

[5] Gupta S., Shrivastava R. M., Tandon R., Gogia V., Agarwal P., Satpathy G. (2011). Role of voriconazole in combined acanthamoeba and fungal corneal ulcer. *Contact Lens and Anterior Eye*.

[6] Schoenwald R. D., Smolen V. F., Bull L. (1985). Controlled drug bioavailability. *Bioavailability Control by Drug Delivery System Design*.

[7] Dupuis A., Tournier N., le Moal G., Venisse N. (2009). Preparation and stability of voriconazole eye drop solution. *Antimicrobial Agents and Chemotherapy*.

[8] Halim Mohamed M. A., Mahmoud A. A. (2011). Formulation of indomethacin eye drops via complexation with cyclodextrins. *Current Eye Research*.

[9] Fridriksdóttir H., Loftsson T., Stefánsson E. (1997). Formulation and testing of methazolamide cyclodextrin eye drop solutions. *Journal of Controlled Release*.

[10] Nijhawan R., Agarwal S. P. (2003). Development of an ophthalmic formulation containing ciprofloxacin-hydroxypropyl-b-cyclodextrin complex. *Bollettino Chimico Farmaceutico*.

[11] Hassan E. E., Gallo J. M. (1990). A simple rheological method for the in vitro assessment of mucin-polymer bioadhesive bond strength. *Pharmaceutical Research*.

[12] Q1A (R2): stability testing of new drug substances and products.

[13] Oviatt H. W., Brant D. A. (1993). Thermal treatment of semi-dilute aqueous xanthan solutions yields weak gels with properties resembling hyaluronic acid. *International Journal of Biological Macromolecules*.

[14] Melton L. D., Mindt L., Rees D. A. (1976). Covalent structure of the extracellular polysaccharide from Xanthomonas campestris: evidence from partial hydrolysis studies. *Carbohydrate Research*.

[15] Paliwal S. K., Chauhan R., Sharma V., Majumdar D. K., Paliwal S. (2009). Entrapment of ketorolac tromethamine in polymeric vehicle for controlled drug delivery. *Indian Journal of Pharmaceutical Sciences*.

[16] Grass G. M., Wood R. W., Robinson J. R. (1985). Effects of calcium chelating agents on corneal permeability. *Investigative Ophthalmology and Visual Science*.

[17] Burgalassi S., Chetoni P., Monti D., Saettone M. F. (2001). Cytotoxicity of potential ocular permeation enhancers evaluated on rabbit and human corneal epithelial cell lines. *Toxicology Letters*.

[18] Burstein N. L. (1984). Preservative alteration of corneal permeability in humans and rabbits. *Investigative Ophthalmology and Visual Science*.

[19] van Horn D. L., Edelhauser H. F., Prodanovich G. (1977). Effect of the ophthalmic preservative thimerosal on rabbit and human corneal endothelium. *Investigative Ophthalmology & Visual Science*.

[20] Malhotra M., Majumdar D. K. (2002). Effect of preservative, antioxidant and viscolizing agents on in vitro transcorneal permeation of ketorolac tromethamine. *Indian Journal of Experimental Biology*.

[21] Maurice D. M., Riley M. V. (1970). Ocular pharmacokinetics. *Biochemistry of the Eye*.

